# Effect of Whole Egg Liquid on Physicochemical, Quality, Fermentation and Sensory Characteristics of Yogurt

**DOI:** 10.3390/foods13020321

**Published:** 2024-01-19

**Authors:** Lijie Yang, Lifeng Wang, Yujie Chi, Yuan Chi

**Affiliations:** 1 College of Food Science, Northeast Agricultural University, Harbin 150030, China; 18663835968@163.com (L.Y.); wanglifeng202312@163.com (L.W.); 2 College of Engineering, Northeast Agricultural University, Harbin 150030, China

**Keywords:** whole egg liquid, fermentation, yogurt, quality

## Abstract

With the purpose of developing an alternative set yogurt with high consumer acceptability, liquid whole egg (LWE), at levels that varied from 0 to 30%, was incorporated into set yogurt, and the effects on the physicochemical, quality, fermentation, and sensory characteristics of yogurt were evaluated. The fat content was lower in egg yogurt than in control yogurt. All color variables were significantly affected by LWE amount. The amount of bacteria in the egg yogurt was greater than in the control yogurt. Sensory analysis data suggested that color, odor, and texture consistently impacted the overall acceptability of the egg yogurt. The addition of 5% whole egg, which resulted in an increase of 6.28-fold in hardness, increase of 6.1-fold in viscosity, decrease in pH values, and a 5.6% decline in water-holding capacity (WHC). The aroma and flavor of the set yogurt was improved as well. LWE addition significantly increased the protein content and dynamic rheology. More importantly, the addition of LWE increased the protein content of the set yogurt. This investigation demonstrated the feasibility of fabricating LWE-enriched set yogurt and its superior quality compared with the corresponding normal product. It also emphasized the reconstruction of LWE with enhanced properties.

## 1. Introduction

Yogurt, a fermented dairy product renowned for its distinct flavor, is produced through the fermentation of milk by lactic acid bacteria. It represents a type of weak protein gel food induced by acidification. Yogurt is highly regarded not only for its nutritional richness and excellent digestibility but also for its potential health benefits, such as reducing blood cholesterol levels, promoting gastrointestinal microecological balance, and strengthening immune function [1]. The research landscape is evolving in response to the changing preferences of consumers toward healthier ingredients and the pursuit of novel and more natural flavors in dairy products. As a result, there is a growing interest in exploring new fermented milk varieties within the field of food technology [2].

Currently, there is a growing interest in foods that offer health benefits beyond their basic nutritional properties. New varieties of yogurt with unique flavors and aromas, which simultaneously offer potential health care functions, have shown increased sales and enhanced consumer satisfaction [3]. Eggs are a significant agricultural product in many countries with enormous annual production [4,5]. On the one hand, eggs are rich in protein, fat, vitamins, and other nutrients [6]. On the other hand, egg proteins have remarkable processing properties such as gelling, foaming, and emulsifying properties, which make them widely used in the food processing industry [7]. In particular, the gelling properties of egg proteins are used in various food products, such as egg tofu and surimi products [8,9]. China’s egg production is substantial, and China has consistently ranked as the top global producer, contributing to approximately 40% of the total egg output worldwide. Therefore, development of the novel deep-processed egg products has wide application prospects. Owing to their substantial protein content (four times greater than that of milk), eggs hold theoretical promise for producing flavored yogurt. Based on this fact, many strategic priorities have been taken into account to increase health-promoting properties to enrich yogurts. More specifically, the addition of flavors and aromas using LWE are used in the development of diversified yogurt with good acceptance, improved nutritional values, and health-promoting benefits.

Owing to multinutritional constituents (including protein, vitamins, minerals, and bioactive compounds) [10], utilizing a combination of eggs and cow milk as raw materials for yogurt production therefore imparts a unique flavor resulting from their combination as well as improved nutritional value. However, eggs have a much higher protein content, ranging from 11% to 13%, and are predominantly composed of ovalbumin, ovotransferrin, and lysozyme, among proteins. Importantly, many of these egg proteins are less resistant to heat and prone to denaturation at high temperatures.

To the best of our knowledge, very little information is presently available about the effects of LWE on the characteristics of yogurt. To fill this knowledge gap and develop a novel, functional yogurt, we investigated the possibility of producing set yogurt using LWE with attention focused on the effects of the added LWE on the physicochemical, quality, fermentation, and sensory characteristics of yogurt.

## 2. Materials and Methods

### 2.1. Materials and Reagents

Fresh Grade A eggs (average weight 45~55 g) were obtained from Shuangcheng County Farm, Heilongjiang Province, China. Superior milk sugar was obtained from Liaoning Zhongguang Jiawei Food Co. The direct-throw starter YO-MIX 883 (containing *Lactobacillus bulgaricus* and *Streptococcus pyogenes* at a ratio of 1:1) was obtained from Danisco Beijing Sharp Vision Science and Trade Co., Bejing, China. All other reagents used in this study were analytically pure and purchased from Harbin Shengda Reagent Company, Harbin, Heilongjiang Province, China. Wanda Mountain pure milk (ultra-high-temperature sterilized milk) was supplied by Heilongjiang Wandashan Linhai Liquid Milk Co., Harbin, Heilongjiang Province, China.

### 2.2. Experimental Methods

#### 2.2.1. Preparation of Egg Lactic Acid Milk

##### Liquid Whole Egg Processing Flow

Specifically, fresh eggs were obtained, and the shells were sterilized in a 75% alcohol solution for 30 min. Then, the mixture was washed and dried, after which the mixture was stirred with a thermostatic magnetic stirrer until a stable liquid formed. Subsequently, the egg solution was homogenized with a T25 high-speed disperser (T25, LabTech, Inc., Beijing, China) at 10,000 rpm for 1 min to form a homogeneous and stable emulsion.

##### Egg Lactic Acid Milk Fermentation Process

A quantity of fresh cow milk was taken and prepasteurized. Subsequently, the homogenized whole egg mixture (0%, 5%, 10%, 15%, 20%, 25%, and 30%) was added to milk in predetermined proportions, and 8% of white sugar was added to the mixture for a second homogenization. After homogenization, the mixture was sterilized at 65 °C for 20 min, immediately cooled to approximately 42 °C, and subsequently transferred to sterile glass vials (height, 7.7 cm; diameter, 6.4 cm). The mixture was subsequently inoculated under aseptic conditions with 0.3% fermenter and subsequently incubated in a constant-temperature incubator at 42 °C until the pH reached 4.6. After incubation, the samples were refrigerated at 4° (refrigerator, CBCD−258WTPZM, Hefei Midea Refrigerator Co., Ltd., Hefei, China) for 12~24 h and then removed for subsequent analysis and measurement.

#### 2.2.2. Fermentation Dynamics

The change in pH value in set yogurt fermentation was determined every 1 h until the pH reached 4.6 ± 0.1. Three parameters, maximum acidification rate (10^−3^/h), the time at which the maximum acidification rate was reached (h), and time required to reach pH 4.6 (h), were used to characterize the process kinetics [11].

#### 2.2.3. Texture

Yogurt texture was analyzed by the backward-extrusion test using the texture profile TA.XT Plus C (TA.XT Plus C, Stable Micro System, Surrey, UK) equipped with a 5 kg load cell. The parameters were modified from the Exponent program template: cylinder probe of 35 mm diameter, test speed 1 mm/s, with 60% strain and surface trigger force 10 g. Firmness (g), consistency (g*sec), and cohesiveness (g) were evaluated using the Exponent program [12].

#### 2.2.4. Color

The sample was placed into a measuring cup and a previously calibrated portable colorimeter was used (CM-600 d, Konica Minolta Holdings, Inc., Tokyo, Japan) to determine color properties; the test was conducted in triplicate at different positions on the flat dish, and the chromaticity data were expressed as L* (luminance), a* (red–green intensity), and b* (yellow–blue intensity) values [13].

#### 2.2.5. Water Holding Capacity

WHC of yogurt sample was calculated according to the process of Ozcan [14]. A 10 mL centrifuge tube was filled with 7.5 g of sample, then centrifuged at 5000× *g* for 20 min (total weight of the yogurt sample was recorded as M). The expelled whey was carefully separated and weighed (S).

$$WHC=\frac{S}{M}\times 100\%$$

#### 2.2.6. Electronic Nose (E-Nose)

The flavor of the yogurt was determined by an electronic nose (e-nose) device (SA402B, INSENT Inc., Fukushima, Japan) according to a previously published method, albeit with slight modifications [15]. A 3.5 g sample of yogurt that had been postcooked for 12 to 24 h was placed in a sample bottle and covered with a cap. The sample was equilibrated at room temperature for 2 h. The e-nose analysis was carried out via a PEN3 portable e-nose assay using the headspace loading method with the needle inserted into the sample vial. The collected data were analyzed by e-nose WinMuster analysis software in triplicate.

#### 2.2.7. Nutritional Value Determination

The nutritional value of the fermented milks was compared by determining the macronutrients (carbohydrates, lipids, and proteins), water and mineral salts [16]. The energy intake was calculated from the energy elements by applying the formulas:

1 g of carbohydrates provides 17 KJ (4 Kcal);
1 g of lipids provides 38 KJ (9 Kcal);
1 g of protein provides 17 KJ (4 Kcal).

#### 2.2.8. Rheological Properties

The rheological properties of yogurt were characterized using a dynamic shear rheometer (HAAKE MARS40, Thermo Fisher, Waltham, MA, USA). Yogurt (15 mL) was transferred into the cup. The shear sweep test was performed from 0.1 s^−1^ to 300 s^−1^ to study the effect of the shear rate on the apparent viscosity of yogurt samples. The frequency sweep test was performed from 0.01 to 10 Hz at a fixed strain of 0.1%. The storage modulus (G′) and loss modulus (G′′) of yogurt samples were determined from the dynamic stress–strain relationships. All measurements were performed at 25 °C [17].

#### 2.2.9. Total Lactic Acid Bacteria and Growth Dynamics

After the sample was fully shaken, 25 mL of the sample was aspirated with a sterile pipette into a sterile conical flask containing 225 mL of saline (with the appropriate number of sterile glass beads prepositioned inside the flask) and shaken thoroughly to make a 1:10 homogenate of the sample. The subsequent process was conducted in accordance with the [18]. National Standard for Food Safety Food Microbiology Test Lactobacillus Test. The relationship between fermentation time and survival was also plotted. During the logarithmic phase, the specific growth rate ($\mu_{G}$), doubling time ($t_{d}$), and multiplication rate (MR) were calculated using the following equations [19]:

(1) $$Specific\;growth\;rate\;\left(\mu_{G}\right)\left(h^{-1}\right)=\left(\ln x-\ln x_{0}\right)/\left(t-t_{0}\right)$$

(2) $$Doubling\;time\left(t_{d}\right)=Ln(2)/\mu_{G}$$

(3) $$Multiplication\;rate\;(MR)\;=\;1/t_{d}$$

where: *x* = amount of growth after *t* time (*t*) and $x_{0}$ = amount of growth at the beginning time ($t_{0}$).

#### 2.2.10. Sensory Evaluation

The scoring criteria were implemented based on Table 1.

At a temperature of 25 °C, sensory analysis was performed in individual booths under white light. Product sensory evaluation was carried out in terms of color, aroma, appearance, body, flavor, texture, and general acceptability based on a 9-point hedonic scale where 1 = dislike extremely, 2 = dislike very much, 3 = dislike, 4 = dislike slightly, 5 = neither like nor dislike, 6 = like slightly, 7 = like, 8 = like very much, and 9 = like extremely. Consumer acceptance was scored by 10 male and 10 female students and faculty members from an appropriate number of samples which were collected and placed in the tray; consumer acceptance was scored by 10 male and 10 female students and faculty members from the School of Food, Northeast Agricultural University (Harbin, China), whose ages ranged from 20 to 36 years [20]. 

#### 2.2.11. Statistical Analysis

Statistical analysis was performed using IBM SPSS Statistics 26 software, and significant differences were calculated using Tukey’s test at the 95% (α = 0.05) level of agreement. Origin 2020 was used for data fitting as well as plotting, and WinMuster was used for e-nose analysis. All experiments were performed in triplicate. The results are expressed as the mean ± standard deviation.

## 3. Results and Discussion

### 3.1. Effect of Egg Addition on the Fermentation Kinetics of Yogurt

**Figure 1:**
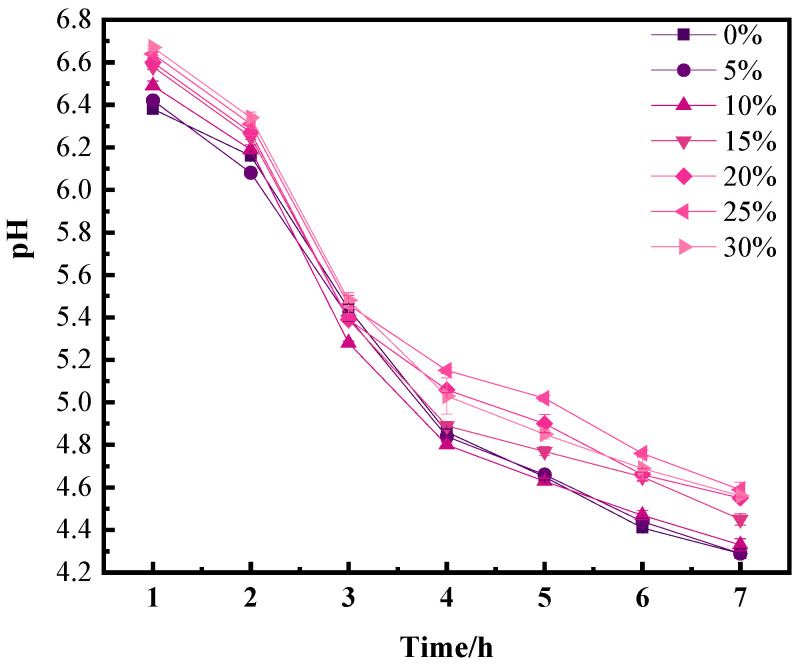
Effect of egg addition on fermentation kinetics of solidified yogurt.

As shown in Figure 1, the effect of the addition of egg liquid to yogurt was explored by monitoring the change in pH during fermentation. Typically, lactic acid is produced by the addition of a fermentation agent, which decreases the pH of all yogurts [21]. Similar results have been found in previous studies [22].

During fermentation, lactic acid bacteria break down lactose to produce organic acids, such as lactic acid, which reduces the pH of the substrate of the egg–milk mixture. The aggregation of proteins is affected mainly by changes in pH and surface charge [23]. Throughout the fermentation process, which spanned from 0 to 7 h, a discernible transition was observed, where the initial egg-lactic mixed fermentation substrate gradually evolved into a weak gel state characterized by a degree of stability. Given that prolonged fermentation can affect product safety, an interval of 0 to 7 h was selected for monitoring fermentation progression and investigating pH variations during the development of the egg-lactic yogurt gel structure. In summary, the pH changes in yogurt augmented with whole egg solution were almost linear. Initially, there was a slight decrease in pH within the first 2 h, followed by a rapid decrease between the hours 2 and 4 and a subsequent smooth and gradual pH change until the fermentation was terminated. For both controls and samples with egg additions ranging from 5% to 30%, the rates of acidification during the initial 1 h were as follows: 2.2 × 10^−1^ pH/h, 3.0 × 10^−1^ pH/h, 3.3 × 10^−1^ pH/h, 3.3 × 10^−1^ pH/h, 3.4 × 10^−1^ pH/h, 3.6 × 10^−1^ pH/h, and 3.7 × 10^−1^ pH/h. Notably, the acidification rate at this juncture exhibited a positive correlation with the proportion of egg solution addition. It should be noted that the pH of the egg solution was initially alkaline; when the egg addition was set at 5%, the time required to reach the fermentation endpoint was the same as that of the control. However, the maximum acidification rate decreased with increasing egg addition, subsequently requiring more time to reach a pH of 4.6 and thereby prolonging the yogurt acidification process. This phenomenon may be attributed to the buffering capacity of the protein in the egg solution.

### 3.2. Effect of Egg Addition on the Texture of Yogurt

**Figure 2:**
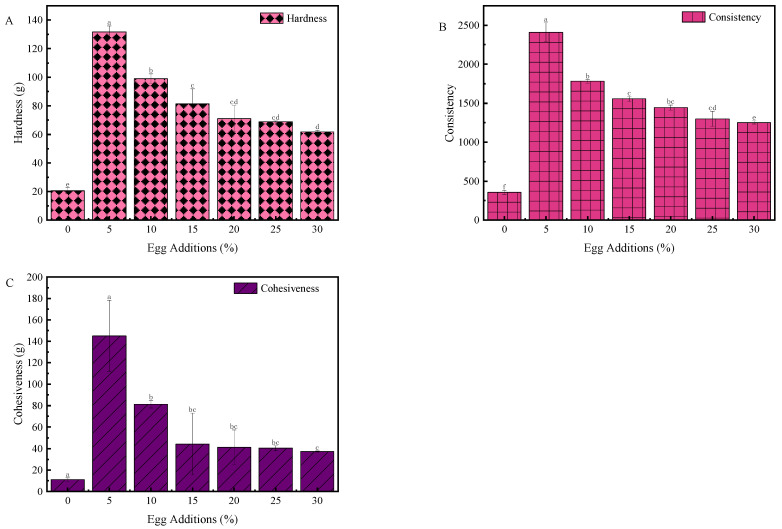
Effect of egg liquid addition on the texture of solidified yogurt: (**A**) hardness (**B**) consistency (**C**) cohesiveness. Different letters indicate significant differences (*p* < 0.05) between the data within each group.

As shown in Figure 2, the effect of egg addition on the texture of yogurt was characterized by measuring the hardness, consistency, and cohesion of the yogurt. The gel structure of yogurt during fermentation and the aggregation of casein micelles due to hydrophobic bonding and electrostatic interactions between k-casein and whey proteins lead to the formation of a three-dimensional mesh structure. The addition of egg solution had a positive effect on hardness, consistency, and cohesiveness [24].

Compared to the control group, the incorporation of egg liquid led to varying degrees of increased yogurt hardness, with the most favorable textural properties observed at 5% egg addition. This augmented hardness was primarily due to the heightened total solids content introduced by the liquid whole egg (LWE) and the interactions between proteins in the LWE and those in milk, resulting in a denser and firmer gel structure [25]. Throughout the fermentation process, proteins undergo a gradual transformation from an initially viscous liquid state to a weak gel state, facilitated by the aggregation behavior driven by diverse intermolecular interactions triggered by pH reduction. The greater firmness exhibited by yogurt is also associated with extended fermentation duration, echoing the patterns observed in fermentation kinetics [26]. Moreover, the addition of the egg mixture resulted in a relatively modest increase in yogurt thickness. Notably, yogurt consistency is intrinsically related to protein content, with higher protein levels contributing to the formation of a more stable structure. Moreover, yogurt enriched with LWE exhibited higher cohesion values than those of the control group, resulting in a smoother internal structure. The observed increased elasticity, indicative of the sample’s ability to revert to its original state following deformation, can also be attributed to the higher water content within the gel, making it softer. This finding suggested that the sample possesses an enhanced capacity to recover its initial position, a trait that aligns with the cohesion results.

### 3.3. Effect of Egg Addition on the Color of Yogurt

**Figure 3:**
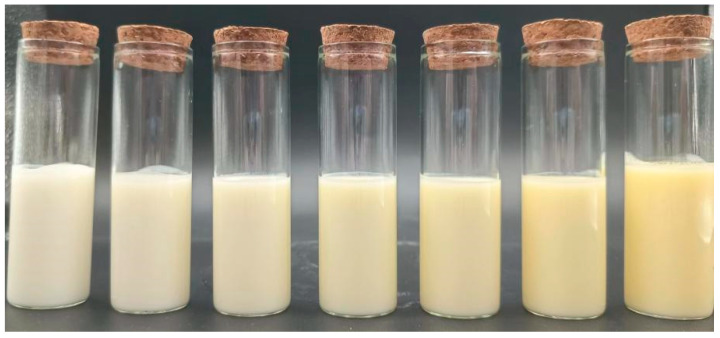
Egg yogurt physical picture.

Color plays a crucial role in influencing consumer acceptance and product marketability in everyday goods, making it one of the most significant visual factors impacting acceptance [27]. The color of sour milk gradually deepens and transitions to a yellow hue, as depicted in Figure 3, in direct correlation with the increasing quantity of LWE added. As shown in Table 2, yogurt samples enriched with a whole egg solution exhibited significantly darker, redder, and yellower hues than the control group (*p* < 0.05). 

This finding is consistent with expectations, considering the inherent coloration of the whole egg mixture (resembling ginger), which imparts a distinctive hue to the yogurt (Table 2). In general, across all yogurt variants, there was a collective increase in redness (a*) and yellowness (b*), accompanied by a reduction in brightness (L*). These trends are likely attributed to the intrinsic properties of the raw materials themselves. Furthermore, of all the yogurt samples, the control sample exhibited the most consistent color attributes, followed by the yogurt sample containing 5% whole egg solution, for which the a* and b* values did not increase significantly (*p* > 0.05). These findings underscore that the introduction of egg solution into yogurt distinctly impacts its color, with the addition of 5% egg solution demonstrating no significant effect (*p* > 0.05) on yogurt redness or yellowness. Notably, the postacidification process of the fermenting agent led to a reduced yogurt pH, which corresponded to an increase in the a* value of the egg-lactic yogurt with prolonged fermentation time. The observed elevation in the yogurt b* values and concomitant decrease in the L* values may be linked to the generation of brown polymers resulting from phenolic degradation (the interaction between PPO and/or POD and phenolic substrates) [28]. In egg-lactic yogurt, the whole egg solution itself possessed favorable emulsifying properties. Conversely, pure milk, a creamy liquid, is brighter than the yellow hue of an egg mixture.

### 3.4. Effect of Egg Addition on the Water Holding Capacity of Yogurt

**Figure 4:**
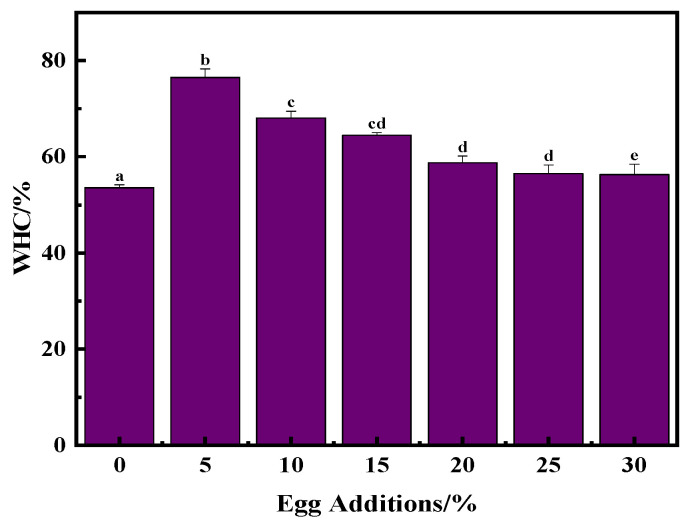
Influence of egg addition on hydraulic retention of solidified yogurt. Different letters indicate significant differences (*p* < 0.05) between the data within each group.

As depicted in Figure 4, the water holding capacity (WHC) was observed to be a pivotal parameter that significantly influenced both consumer acceptance and the shelf life of yogurt [29]. Stabilizing complexes characterized by robust internal bonds have been reported to mitigate protein rearrangements. These complexes play a critical role in sustaining the three-dimensional network formed by casein, thereby maintaining hydration levels within the yogurt system and reducing dehydration [12].

The influence of egg addition on the moisture content of yogurt is evident from the figure. Relative to that of the control group, the WHC exhibited a gradual increase with the progressive inclusion of egg liquid, ultimately surpassing that of the control group when 30% whole egg mixture was added. Notably, the WHC of the control group was significantly lower than that of the experimental groups (*p* < 0.05). This difference underscores how the reduction in milk fat content correlates with a decrease in yogurt WHC. Indeed, empirical evidence confirms that, with identical yogurt recipes and fermentation conditions, the WHC of yogurt diminishes with decreasing fat content, as confirmed by texture measurements of yogurt with varying fat levels. A low WHC was achieved when 5% whole egg solution was added. Samples with this addition level exhibited superior water retention capabilities, yielding the most stable system. This phenomenon can be attributed to the increase in intermolecular and intramolecular hydrogen bonding, which results in a tighter network structure, substantially increasing the WHC of yogurt. However, with extended fermentation, the pH of the yogurt decreased below the isoelectric point of the proteins, leading to an accumulation of positive charges on the proteins, which triggers repulsive interactions between the proteins, thereby somewhat diminishing the WHC of yogurt. Consequently, there was a decrease in WHC over time, although it generally remained higher in the experimental groups than in the control group. Additionally, at the beginning of fermentation, various interactions, such as ionic bonding and hydrogen bonding, occur between water molecules and protein molecules. However, as fermentation progresses, water molecules become essentially entrapped within the gel network structure. Importantly, this portion of water molecules is not stable and tends to separate easily when the gel structure is disrupted or subjected to external forces. Previous research has indicated that stable complexes can form between phenolics and casein, thereby accommodating a greater volume of yogurt whey [30].

### 3.5. Effect of Egg Addition on Yogurt Odor

**Figure 5:**
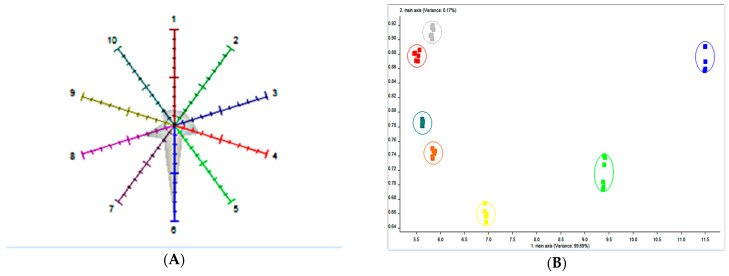
An electronic rhinogram: (**A**) radar graph and (**B**) PCA chart. Note: 1 represents W1C, 2 represents W5S, 3 represents W3C, 4 represents W6S, 5 represents W5C, 6 represents W1S, 7 represents W1W, 8 represents W2S, 9 represents W2W, and 10 represents W3S.

Flavor is a pivotal quality attribute that profoundly influences the acceptability of yogurt among diverse consumer groups [31]. To explore the impact of whole egg solution addition on yogurt aroma, an electronic nose analysis was conducted on the yogurt samples, as depicted in Figure 5A,B. Figure 5A portrays the aroma characteristics of the yogurt samples after the addition of various amounts of LWE. Across all the yogurts, W1C (aromatic compounds, primarily associated with flavor), W5C (alkanes), and W3S (aromatic alkanes) displayed elevated response values. These findings suggested that the corresponding flavors represented by these sensors are the primary contributors to yogurt flavor. Notably, yogurt samples augmented with whole egg solution exhibited notable differences.

Higher response values were observed for W1S (methyl species) and W2W (organic aromatic sulfides). The response values of these sensors (W1S, W2W) increased with increasing whole egg solution addition, indicating that the inclusion of the whole egg mixture augmented the aromatic compounds within the yogurt. The heightened signals of these sensors may be linked to both the inherent odor of the whole egg solution and the aromatic substances generated during LAB fermentation. Figure 5B shows the principal component analysis (PCA) of yogurt samples treated with various concentrations of added whole egg solution. The first two principal components (PCs) accounted for 99.86% of the total data variance (PC1: 99.69%, PC2: 0.17%), which surpassed 80% and therefore adequately captured the essential information from the original high-dimensional data matrix. These two PCs encapsulate the key information characteristics of the samples, offering a representative overview of the overall characteristics of yogurt. In a general sense, most yogurt samples can be broadly categorized into clusters located in four distinct regions. It is evident from the figure that yogurt samples with and without whole egg solution addition occupies separate regions. A pronounced separation is observed, particularly with the control samples positioned significantly apart from the experimental samples. This substantial difference highlights the discernible shift in yogurt flavor caused by the introduction of the whole egg solution. Among them, blue represents 0%, bright green represents 5%, red represents 10%, gray represents 15%, yellow represents 20%, dark green represents 25%, brownish-yellow represents 30%. Notably, yogurt enriched with 5% whole egg liquid closely aligned with the control group, signifying a similarity in aroma between the two groups. Conversely, samples containing 10%, 15%, 25%, or 30% whole egg liquid were combined. This clustering indicates that these samples share comparable aroma profiles, underscoring that the addition of whole egg solution enhances the aroma of yogurt.

### 3.6. Nutritional Value of the Fermented Milks

The nutritional value of the different fermented milk samples was determined, and the results are shown in Table 3. The nutritional analyses showed that the carbohydrate content of the samples slightly differed. 

This difference may be attributed to the very small amounts of polysaccharide substances, such as beta-glucan and starch, in eggs and milk; as expected, these substances are not present in eggs or milk alone [32]. On the other hand, protein content was greater in yogurt containing whole egg solution than in plain yogurt, for which a protein content of 3.3 g/100 g was recorded. This was due to the high protein content of the eggs, which was 11% to 13%. High carbohydrate and fat contents contributed to the high energy content of plain yogurt (362.0 kJ/85.4 kcal). Consequently, high protein and low fat levels are more suitable for individuals who suffer from obesity and cardiovascular diseases, as the energy level is lower. Moreover, low protein levels in people who do not have sufficient access to protein-rich foods are detrimental to their overall health. It is therefore necessary to produce protein-enriched products by adding protein extracts and high-protein raw material, such as chickpea, soy protein, and egg protein, to improve protein intake.

### 3.7. Effect of Egg Addition on the Rheological Properties of Yogurt

#### 3.7.1. Apparent Viscosity

**Figure 6:**
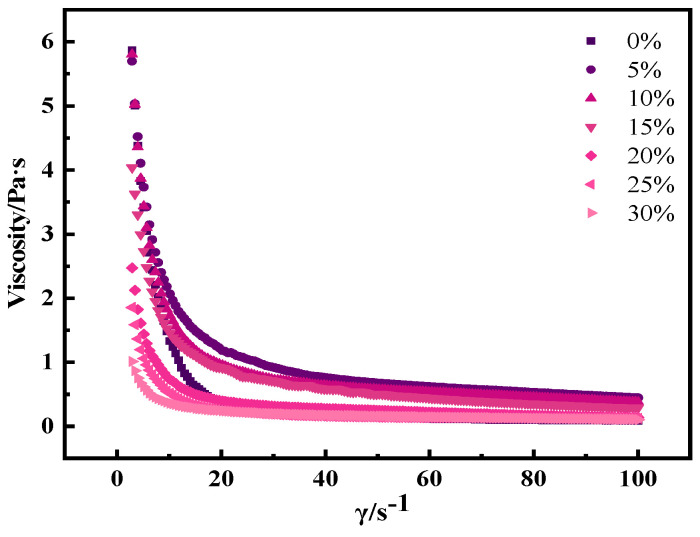
Effect of egg liquid addition on the apparent viscosity of solidified yogurt.

The apparent viscosity is defined as the ratio of the shear stress to the rate for a specific velocity gradient. Previous research has indicated that yogurt products, particularly those high in protein, often exhibit non-Newtonian fluid behavior [33,34]. As illustrated in Figure 6, yogurt enriched with whole egg solution exhibited shear-thinning behavior, underscoring its non-Newtonian fluid nature. This characteristic arises from the formation of a more stable three-dimensional network structure among particles, which hinders their flow and imparts a certain level of viscosity.

In comparison, the control yogurt exhibited a relatively low initial apparent viscosity, which increased with the addition of whole egg solution. The highest apparent viscosity was observed in samples containing 5% whole egg solution, which was indicative of enhanced stability. Notably, the initial apparent viscosity did not fully recover when the shear rate decreased from 100 to 0.1 s^−1^ during downward scanning. This phenomenon suggested some structural damage within the yogurt, potentially related to the fat content of the ingredients. Research has shown that gel systems containing fat often exhibit elevated apparent viscosities [35]. In the protein gel network structure, fat particles can aggregate and fill spaces, thereby altering the rheological properties of yogurt. The addition of the whole egg mixture led to an increase in the apparent viscosity of the yogurt. This may be attributed to several factors: first, the replacement of some milk with egg, resulting in increased fat content, which impedes flow behavior [36]. Second, alterations in the particle charge due to egg addition lead to heightened interparticle repulsion, increased flow resistance, and the formation of a stable system.

#### 3.7.2. Elastic Modulus and Viscous Modulus

**Figure 7:**
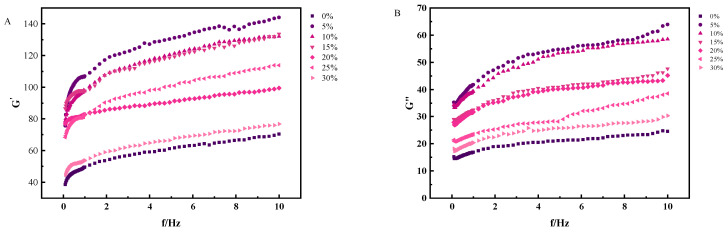
Effect of egg liquid addition on the (**A**) energy storage modulus and (**B**) loss modulus of solidified yogurt.

As depicted in Figure 7, the values of G′ and G″ for all the yogurt samples increased slightly with increasing angular frequency. 

Furthermore, the G′ values consistently surpassed the G″ values, confirming the solid-like behavior of the yogurt. Moreover, the incorporation of the whole egg mixture led to a modest reduction in both the G′ and G″ values compared to those of control yogurt. However, these values remain higher than those of the control group. This observation implied that the addition of the whole egg solution enhanced the aggregation behavior of the yogurt, resulting in decreased fluidity and a transition toward a semisolid or solidified state. This, in turn, contributed to the formation of a more tightly interconnected network structure, ultimately leading to an increase in G′. Consequently, this increase in G′ enhanced the stability of the yogurt system. In essence, these findings underscore the beneficial impact of incorporating whole egg solution into yogurt formulations.

### 3.8. Total Lactic Acid Bacteria

**Figure 8:**
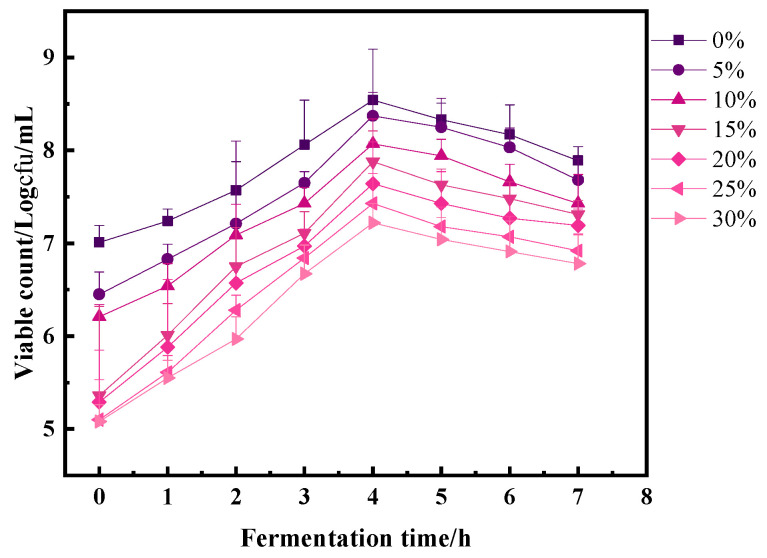
Effect of egg liquid addition on the number of viable bacteria.

As depicted in Figure 8, the viability of lactic acid bacteria in egg-lactofermented yogurt significantly increased (*p* < 0.05) from the initial stages of fermentation until the 7th hour. The highest count of viable bacteria within the yogurt was recorded at the 4th hour of fermentation. Notably, as the quantity of whole egg solution added increased, the number of live bacteria slightly decreased, although this difference was not statistically significant.

The bacterial counts at different levels of whole egg solution addition were as follows: 7.89 $\times \;10^{7}cfu/mL$, 7.68 $\times \;10^{7}cfu/mL$, 7.43 $\times \;10^{7}cfu/mL$, and 7.31 $\times \;10^{7}cfu/mL$, 7.19 $\times \;10^{7}cfu/mL$, 6.92 $\times \;10^{7}cfu/mL$, and 6.78 $\times \;10^{7}cfu/mL$. The highest count of lactic acid bacteria was observed in the 5% whole egg solution treatment group compared to the other experimental groups. Furthermore, the growth of lactic acid bacteria remained stable until the conclusion of the fermentation process. The growth parameters of the yogurt cultures were measured during the logarithmic growth curve. The specific growth rates ($\mu_{G}$) were 0.0168 h^−1^, 0.0249 h^−1^, 0.0256 h^−1^, 0.0443 h^−1^, 0.0438 h^−1^, 0.0435 h^−1^, and 0.0412 h^−1^, while the multiplication times ($t_{d}$) were 4.78, 4.37, 4.38, 3.81, 3.82, 3.83, and 3.88 for the experimental conditions. Additionally, the multiplication rates (MRS) were determined to be 0.209, 0.228, 0.228, 0.262, 0.261, 0.261, and 0.258. These growth parameters provide valuable insights into the dynamics of lactic acid bacteria during fermentation.

Lactobacillus fermentation represents a straightforward and secure method of preservation. The incorporation of probiotics into yogurt can yield dairy products distinguished by their distinctive flavors, textures, and associated health benefits. Furthermore, as highlighted in the study by de Mesquita et al. [37]., Lactobacilli cultured in De Man, Rogosa and Sharpe (MRS) medium exhibited specific growth rates ranging from 0.12 h^−1^ to 0.21 h^−1^ and multiplication times ranging from 1.38 h to 2.44 h. The impact of whole egg solution addition on the growth activity of lactic acid bacteria during yogurt fermentation aligned with the observed changes in pH during this process. It can be postulated that the presence of whole egg solution moderated the pH shift in yogurt by influencing the growth of lactic acid bacteria throughout fermentation. This impact on lactic acid bacteria growth may be attributed to the insufficient carbon source within the whole egg mixture, which is notably rich in proteins but extremely low in carbohydrates, necessitating external supplementation from other sources [38]. It is crucial to recognize that postacidification of yogurt is one of the pivotal factors influencing its shelf-life quality. In the context of the present study, preliminary evidence suggested that whole egg solution inhibits the growth of both *L. bulgaricus* and *S. thermophilus*. Consequently, leveraging this property to mitigate postacidification in yogurt holds significant potential.

### 3.9. Sensory Evaluation

**Figure 9:**
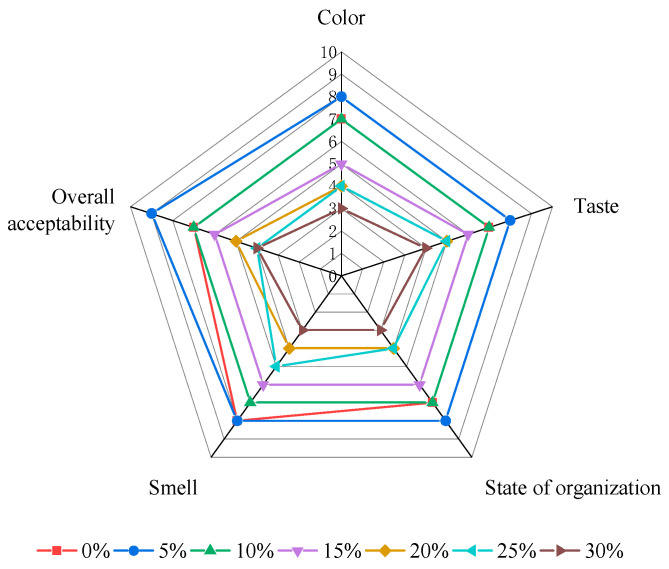
Egg-lactic milk sensory score.

The sensory evaluation scores of yogurt samples containing whole egg at various levels are presented in Figure 9. 

In terms of texture, samples with 30% whole egg addition exhibited poor coagulability and obtained the lowest score. The sensory scores were highest when the whole egg addition level was 5%. The samples also demonstrated inferior scores in terms of taste and overall acceptability, with the 30% whole egg addition group falling below the other experimental groups. However, in terms of flavor, no significant differences were observed among the other experimental groups, potentially because the 30% whole egg addition samples exhibited inadequate solidification but possessed a delicate taste.

### 3.10. Correlation Analysis

**Figure 10:**
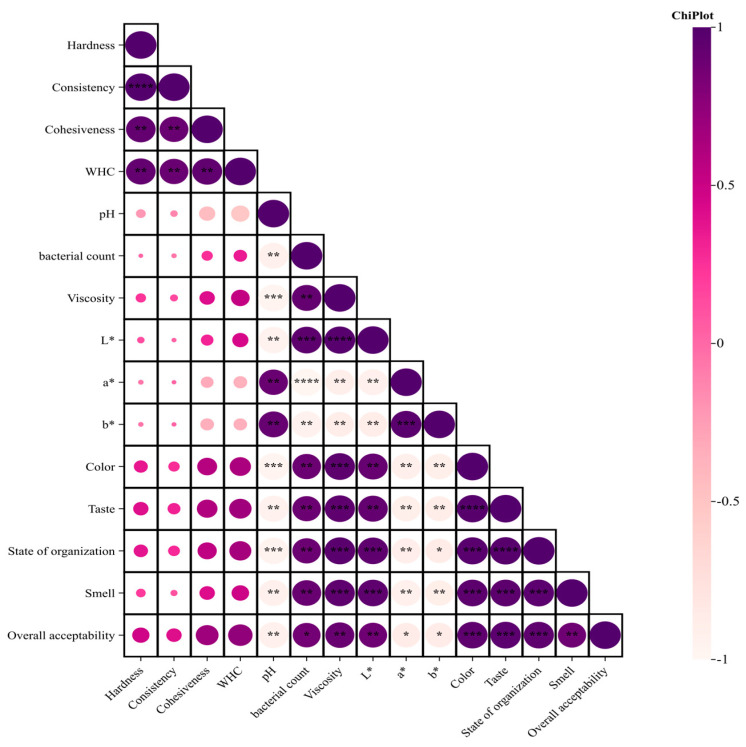
Correlation of egg milk fermented yogurt. Note: * indicates significant, ** indicates extremely significant, *** indicates very significant, and **** indicates super significant.

Figure 10 shows the Pearson correlation coefficients among the variables studied in egg yogurt. A positive correlation indicates mutual promotion, while a negative correlation suggests reciprocal inhibition. Overall acceptability was significantly positively correlated with smell and extremely significantly positively correlated with state of organization, taste, and texture, indicating that in addition to taste, the other three are key sensory indicators of yogurt. The texture of yogurt is an important factor affecting its taste, and the texture is positively correlated with the WHC. The pH correlated negatively with the number of living bacteria. This is because the strain utilizes sugars during yogurt fermentation, and an increase in lactic acid bacteria leads to greater acid production and a lower pH [13]. Additionally, L*, a*, and b* are mutually inhibited by the number of viable bacteria and viscosity but mutually promoted by pH. Correlation analysis revealed the intrinsic relationships between different indicators of yogurt quality, which can improve yogurt quality control measures.

## 4. Conclusions

Adding different amounts of whole egg solution affects the fermentation and quality compared to those of control yogurt differently. In the present study, when the concentration of whole egg liquid added to yogurt ranged from 0% to 30%, the organoleptic quality was good, and the fermentation process was somewhat affected as the amount of egg mixture gradually increased. As the fermentation time subsequently increased, the stability of the gel network structure and the textural properties of the yogurt improved, the pH of the egg yogurt decreased. The WHC also first increased and then decreased, but it was greater than that of the control group. Moreover, the addition of whole egg solution increased the protein content of the yogurt. The rheological results revealed significant changes in G′ and G″ values and reduced fluidity, which improved the stability of the yogurt system. In summary, the moderate addition of whole egg solution improved the fermentation and quality of yogurt, confirming and it can be used in the production of yogurt. Therefore, in the food industry, the addition of whole egg liquid is of practical significance for the development of new zero-additive, whole-protein yogurts. The results of the present study indicate that it is feasible to add whole egg solution to yogurt, broaden the range of flavored yogurts, and expand the use of whole egg liquid. This is evidence that the enrichment of natural yogurt with LWE yields an innovative fermented product with improved nutritional value.

## Figures and Tables

**Table 1:** Sensory evaluation form.

Evaluation Index	Evaluation Criteria	Total Score (100 Points)
Color (10 min)	The color is mottled and chromatic	0~4
	The color is more uniform, and the color is better	5~7
	The color is uniform, and the color is good	8~10
Taste (10 points)	The taste is rough and grainy	0~3
	The texture is smooth and light	4~6
	The taste is fine and not grainy	7~10
State of organization (10 points)	There are pores on the surface, and the whey precipitation is obvious	0~4
	There are fewer pores and whey precipitation	5~7
	No porosity and whey precipitation	8~10
Smell (10 points)	There is a distinct odor	0~3
	Lighter odor	4~6
	The egg aroma is obvious	7~10
Overall acceptability (10 points)	Dislike extremely	0~3
	Like	4~6
	Like extremely	7~10

**Table 2:** Influence of the amount of egg liquid on color difference of solidified yogurt. Different letters indicate significant differences (*p* < 0.05) between the data within each group.

Whole Egg Additions	L*	a*	b*
0%	101.23 ± 0.34 ^a^	−1.08 ± 0.04 ^a^	12.74 ± 0.02 ^b^
5%	99.91 ± 0.03 ^c^	−0.78 ± 0.02 ^a^	14.35 ± 0.07 ^a^
10%	99.27 ± 0.39 ^c^	0.07 ± 0.05 ^d^	19.95 ± 0.04 ^d^
15%	98.22 ± 0.16 ^b^	0.46 ± 0.05 ^a^	24.52 ± 0.15 ^a^
20%	94.75 ± 0.53 ^a^	0.60 ± 0.03 ^b^	24.88 ± 0.39 ^b^
25%	93.83 ± 0.09 ^b^	1.89 ± 0.02 ^c^	26.17 ± 0.18 ^a^
30%	92.28 ± 0.24 ^a^	1.44 ± 0.16 ^a^	26.93 ± 0.89 ^c^

**Table 3:** Nutritional value of fermented milks per 100 g of products.

Yogurt Samples	Nutrient Composition				
	Proteins (g)	Fat (g)	Carbohydrates (g)	Energy (kJ)	Energy (kcal)
0%	3.3	3.8	9.5	362.0	85.4
5%	7.0	3.3	4.5	320.9	75.7
10%	8.4	3.6	4.2	351.0	82.8
15%	9.1	4.0	4.0	374.7	88.4
20%	10.0	4.5	4.2	412.4	97.3
25%	11.6	4.7	5.4	467.6	110.9
30%	12.1	5.1	6.3	506.6	119.5

## Data Availability

Data is contained within the article.
